# The Common bed bug (*Cimex lectularius*) in metropolitan France. Survey on the attitudes and practices of private- and public-sector professionals

**DOI:** 10.1051/parasite/2016038

**Published:** 2016-09-08

**Authors:** Frédéric Jourdain, Pascal Delaunay, Jean-Michel Bérenger, Yvon Perrin, Vincent Robert

**Affiliations:** 1 Centre National d’Expertise sur les Vecteurs, Centre IRD France Sud 911 avenue Agropolis BP 64501 34394 Montpellier cedex 5 France; 2 Service de Parasitologie-Mycologie, Hôpital de l’Archet, Centre Hospitalier Universitaire de Nice 06000 Nice France; 3 Inserm U1065, Centre Méditerranéen de Médecine Moléculaire, Université de Nice-Sophia Antipolis 06000 Nice France; 4 Unité de Recherche en Maladies Infectieuses Tropicales (URMITE), UM63, CNRS 7278, IRD 198, Inserm 1095, Faculté de Médecine de la Timone, Université Aix-Marseille 13000 Marseille France; 5 UMR MIVEGEC, IRD 224-CNRS 5290-UM, Centre IRD France-Sud 911 avenue Agropolis BP 64501 34394 Montpellier France

**Keywords:** Bed bug, Municipal Health and Safety Services (MHSSs), Pest management companies (PMCs)

## Abstract

The Common bed bug, *Cimex lectularius,* had virtually disappeared from France in the 1950s; however, a worldwide resurgence of bed bugs (*C. lectularius* and *C. hemipterus*) has been observed since the 1990s. To document modern pest control activities for the management of bed bugs, a survey was conducted in metropolitan France among the two main categories of professionals regularly called upon to deal with the control of infestations: Municipal Health and Safety Services (MHSSs) and private Pest Management Companies (PMCs). These professionals responded to a questionnaire targeting their knowledge, attitude and practices related to the process for diagnosing a bed bug infestation and the processes taken to actually control an infestation. There were 68 responses received from MHSSs and 51 from the PMCs. The responses indicate that every single *département* (French administrative division) in metropolitan France has witnessed at least one intervention for bed bugs. Among the criteria considered sufficient to confirm a bed bug infestation, direct observation of bugs was the most commonly cited response. Faced with an infestation, most PMCs used a combination of non-chemical and chemical methods, and systematically performed two treatments. This survey is the first of professionals involved in bed bug control in metropolitan France and confirms the growing importance of bed bugs as a public health pest. Establishing a database to monitor this emerging pest would improve the understanding of the distribution of these insects, help guide educational requirements, identify research needs and assist in ensuring that the most appropriate control practices are undertaken.

## Abstract (fr)

La punaise de lit *Cimex lectularius* avait pratiquement disparu en France dans les années 1950, mais une recrudescence mondiale des punaises de lit (*C. lectularius* et *C. hemipterus*) a été observée depuis les années 1990. Une enquête a été conduite en France métropolitaine pour documenter les activités de lutte parmi les deux catégories de professionnels régulièrement appelés en cas d’infestations par punaises de lit: les Services Communaux d’Hygiène et de Santé (SCHS) et les entreprises privées de Dératisation, Désinfection et Désinsectisation (entreprises dites “3D”). Ces professionnels ont répondu à un questionnaire visant leur connaissance, attitude et pratiques apportées au processus de diagnostic d’une infestation par punaises de lit et aux actions de lutte entreprises. Les réponses ont été au nombre de 68 pour les SCHS et de 51 pour les 3D. Il apparaît que chaque département de France métropolitaine a fait l’objet d’au moins une intervention contre des punaises de lit. Parmi les critères considérés comme suffisants pour confirmer une infestation, l’observation directe de la punaise de lit était la réponse la plus fréquente. Pour lutter contre une infestation, la plupart des 3D utilisent une combinaison de méthodes non-chimiques et chimiques et exécutent systématiquement deux traitements. Cette enquête est la première réalisée auprès de professionnels impliqués dans le contrôle de l’infestation en France métropolitaine. Elle confirme l’importance croissante des punaises de lit en tant que problème de santé publique. Il semble souhaitable de mettre en place une base de données afin d’améliorer notre connaissance de la distribution de ces insectes, d’aider à la formation, d’identifier les recherches nécessaires, et d’encadrer de façon optimale les pratiques de lutte.

## Introduction

The Common bed bug, *Cimex lectularius*, had virtually disappeared from France in the 1950s, probably as a result of an improvement in the general hygiene of housing and the effective use of DDT and other organochlorines [14]. However, this situation did not last as in the early 1990s, a resurgence of bed bugs was observed in most nations, including France [1, 6]. The global resurgence has involved two species, *C. lectularius* and *C. hemipterus* [12]. The latter may be imported by travellers on a regular basis from tropical to temperate regions, but the winter months in France are likely too cold for the species to become established. The prevalence of *C. hemipterus* in France is much lower than that of *C. lectularius* [5], but precise records are lacking, as identifications of bed bugs to species level are not routinely undertaken. The reasons for the resurgence are somewhat contentious, however insecticide resistance appears to be the main factor along with the increase in international transport through tourism and trade [4, 10].

In terms of human health, there are as yet no proven cases of pathogens being transmitted to human beings by *Cimex*, although laboratory experiments suggest that vector-borne transmission of some pathogens, such as *Trypanosoma cruzi*, could be a possibility [11, 18]. To date, despite millions of people being bitten every day worldwide, there is no evidence of transmission in the field. Despite this, cutaneous reactions to bites are common and well documented [12]. The psychological impacts of bed bug infestations are significant to the individual, causing serious distress [3, 9, 13], which is often coupled with major financial costs as bed bug control is expensive (although poorly documented). Bed bugs have thus become a serious public health nuisance.

From a health viewpoint, bed bugs are considered as a mere nuisance in France; as such, they are not recognised as a major public health problem and are not a priority for the health authorities. At present, the problem is the responsibility of mayors, as part of their health and hygiene brief, and of property owners, or occupants. In other words, in France, the state health authorities have no legislative responsibility for bed bugs; rather, it is the local authorities at the municipal level that are responsible. Under the current legislation, the costs of insect control are to be borne by the tenants, unless they can prove that the bed bugs were already present before they moved in. In this case, responsibility passes to the landlord, who, under French law, has an obligation to provide a safe and habitable dwelling.

When it comes to bed bug control, there is no such thing as a tolerable level of infestation. Once an infestation has been detected, the aim is to totally eradicate all the bed bugs present. Coupled with resistance and the lack of efficacious products, bed bug control is acknowledged to be difficult, particularly in multiple occupancy dwellings (e.g. apartment buildings, hotels, retirement homes, prisons, etc.). The fact that multiple stakeholders are involved in bed bug control introduces further complexity. The Bed Bug Foundation (www.bedbugfoundation.org) established the European Bed Bug Code of Practice in 2011 with a second edition in 2013. To date, this has yet to be translated into French and thus presently does not constitute a tool used by professionals. The lack of data on professionals faced with the challenge of bed bug control in metropolitan France has caught the attention of a number of national experts.

Taking account of the worldwide resurgence in bed bugs, a survey was undertaken on the impact of the resurgence on pest control professional activities in France, especially among the two main groups of professionals: public health services (primarily Municipal Health and Safety Services, MHSSs) and private pest management companies (PMCs). The first objective was to document the working methods, attitudes and management practices. A secondary objective was to compare the responses of these two professional groups.

## Materials and methods

### The expert group

The French National Centre of Expertise on Vectors (CNEV), which reports to the Ministry for Health and Agriculture, set up a working group on bed bugs in metropolitan France in 2013. The conclusions of this expert group are now available in the form of a downloadable report [5] and a booklet aimed more specifically at the general public facing bed bug concerns [7]. The expert group undertook the present survey in the framework of the CNEV’s activities.

### Municipal Health and Safety Services (MHSSs)

Municipal or Inter-municipal Health and Safety Services are responsible for applying environmental and health protection provisions that fall within the remit of municipal authorities. Their organisation and financing are the responsibility of the mayor, or the president of a public institution for inter-municipal cooperation (association of local authorities). Some services also, by way of exception, perform duties normally performed by the State, including the administrative and technical monitoring of hygiene rules, and particularly substandard housing. Within this framework, the MHSSs may also be called upon in the event of a bed bug infestation. Depending on the case, the MHSSs may intervene by providing information and advice, taking preventive, protective, educational or health promotion measures, or by legally enforcing the legislation. In almost all cases, the MHSSs do not play an operational role in bed bug control. When bed bugs are discovered, in a private residence or in social housing, for instance, the MHSSs do not treat the infestation. A private PMC takes over at that stage. Some MHSSs have a pest control centre that intervenes, at the request of residents, as a service provider in the treatment of insect infestations with a public health impact (cockroaches, fleas, bed bugs, mosquitoes, urticating caterpillars, hornets, etc.).

The total number of Municipal Health and Safety Services (MHSSs) and similar services in France is 208 [16].

### Pest Management Professionals (PMPs) and Companies (PMCs)

PMPs are grouped into private PMCs which intervene at the request of local authorities, private individuals and businesses. PMPs perform a diagnosis based on detecting animals or traces of animals and carry out localised or more extensive interventions (throughout an apartment building, for instance) to treat infestations. It may perform periodic inspections or take preventive actions. The size of PMCs can vary hugely. Most of them have between one and three employees, but those that operate nationwide can have several hundred staff. These companies are governed by a national collective pay agreement. They require authorisation to operate commercially. Any person (user, distributor or purchaser of certain biocidal products) handling biocidal products for professional use must hold a mandatory individual certificate accrediting him/her as a “professional user and distributor of certain kinds of biocidal products intended exclusively for professional use”, commonly referred to as “Certibiocide”. This system aims to ensure that professionals using such products are competent, given the potential risks the products pose for the general public, the environment and the users themselves. This certificate is obtained after 21 h’ (3 days’) training with a training organisation accredited by the Ministry in charge of environmental issues.

### The questionnaire

The survey questionnaire (Supplementary Material) was composed of seven sections:

1. Identification of the respondent.
2. Characteristics of the company (for PMCs only).
3. Volume of activities concerning bed bugs.
4. The bed bug diagnosis procedure.
5. Actions taken to control bed bugs (for PMPs only).
6. Training/information needs.
7. An open section for any other comments.

### The survey

The questionnaire was a retrospective survey focusing on operations in 2014.

The responses were provided by the head of the service or the technician responsible for bed bug control operations.

The MHSSs were contacted only by email to ask them to complete the questionnaire.

PMCs were approached in three stages, in an effort to increase the number of responses to the questionnaire:

- The first call for participants took the form of an advertisement in the trade press (the magazine NPI – *Nuisibles et Parasites Information*) [2];
- The second call for participants was sent by email in late 2013, with a reminder in March 2014, to the mailing list of trade magazine NPI, which comprised around 3200 addresses;
- A third approach was made by telephone to companies affiliated with the main PMC association. There are 179 such companies (http://www.cs3d.info/liste-des-adherents/) (between May and November 2014). Through this method, 108 companies spread across all the *départements* of metropolitan France were contacted.

The results were compiled and analysed using Microsoft Excel. When appropriate, Student’s *t* test and Fisher’s exact test were used to compare the responses of MHSSs and PMP. A significance threshold of 5% was used for these tests.

### Ethical considerations

A declaration file was forwarded to the *Commission nationale de l’informatique et des libertés* (CNIL) which ensures the protection of personal data (declaration registered under No. 1979826v0).

## Results

### Number of respondents

Out of the 208 MHSSs contacted, 68 answered the questionnaire.

Out of the 108 PMPs contacted, 51 answered the questionnaire. The first call for participants in the trade press yielded nine responses. The second call for participants (by email) did not yield any responses. The third approach (by telephone) was more successful, yielding 42 responses. Non-respondents (108–42 = 66) could be broken down as follows: 55 no-replies for unknown reasons, 5 rodent control companies that do not carry out insect control, 3 firms that distribute pest control products but do not themselves carry out treatment, 2 training/education institutions that do not carry out treatment and 1 that could not be reached by phone or email (Table 1).

**Table 1.** Bed bug surveys sent to Municipal Health and Safety Services (MHSSs) and private Pest Management Companies (PMCs). Data collection methodologies and number of responses to the questionnaire; NA = not applicable.

	MHSSs	PMC
Number in metropolitan France	208	179*
Call for participants in trade magazines	208	179
Number of responses	68	9
Approached by email	NA	3200**
Number of responses	NA	0
Contacted by telephone	NA	108
Number of telephone responses	NA	42
Total responses: 119	68	51

* PMCs affiliated with the main PMC association (see Materials and Methods).

** This large list of email addresses targets people working in France in the PMCs (several entries can exist for the same company) and other professionals not necessarily involved in bed bug related activities including manufacturers, distributors and end users of biocidal products, such as agribusinesses, mass-market retailing and transport companies.

All *départements* in metropolitan France responded.

### Prevalence of bed bug call-outs

Eighty-five per cent (85%) of MHSSs (58/68) and 100% of PMCs (51/51) said they had been called out to deal with bed bugs in the past.

Ordinarily, MHSSs do not take any action against bed bugs, leaving this task to PMCs.

For PMCs, bed bug control activities account for:

- less than 20% of total business for 80% of respondents (39/49);
- 20–40% of total business for 18% of respondents (9/49) and;
- 40–60% of total business for 2% of respondents (1/49) (total = 100%).

In terms of the number of interventions per year: 2% of respondents reported no interventions; 22%, fewer than 10 interventions; 44%, 11–50 interventions; 20%, 50–100 interventions; 4%, 140–200 interventions; and 8%, more than 200 interventions (*n* = 50).

### Assessment of infestations in metropolitan France

Based on the declarations by the MHSSs and the PMCs, at least one intervention against bed bugs was reported in every *département* of metropolitan France.

### Change in the number of bed bug call-outs

For MHSSs: 50% (34/68) consider that the number of call-outs has increased over the past five years, 15% (10/68) think it has remained stable, and 3% (2/68) believe it has fallen, and 32% (22/68) did not say.

For PMCs: 80% (41/51) consider that the number of call-outs has increased over the past five years, 18% (9/51) think it has remained stable, no respondents believed that it had fallen (0/51) and 2% (1/51) did not say.

**Figure 1.**
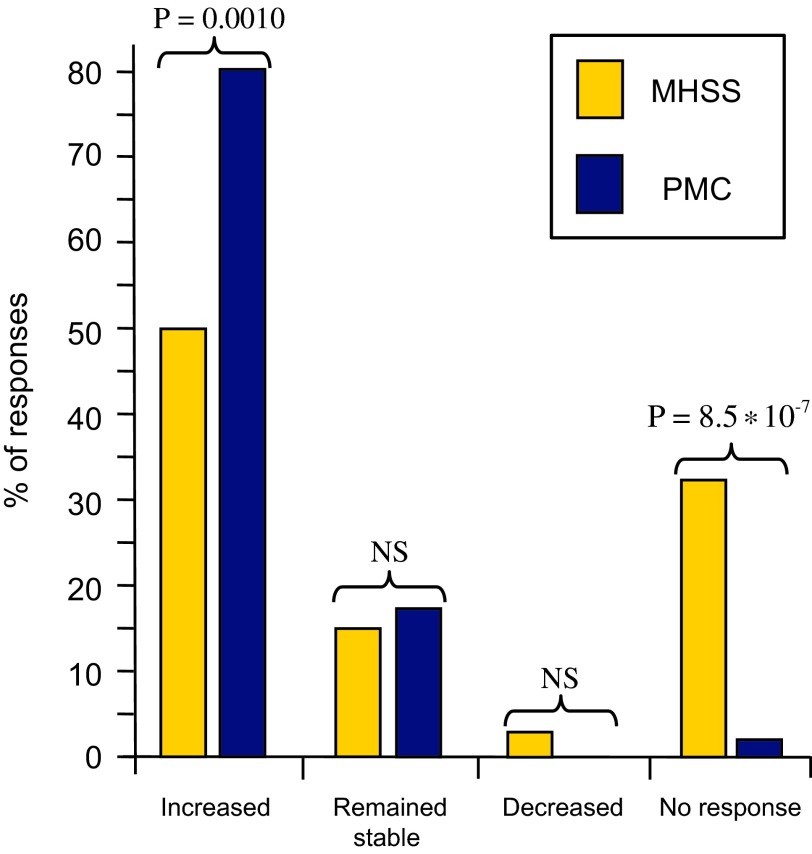
Percentage of responses from Municipal Health and Safety Services (MHSSs) and Pest Management Companies (PMCs) regarding their perception of the evolution of the bed bug problem over the past 5 years. Fisher’s exact probability (P) is indicated when significant; NS: Not significant.

A comparison of the responses given by MHSSs and PMCs indicates significant differences in the responses on growth and on failure to reply (Fig. 1).

### Criteria thought sufficient (knowledge) for determining an infestation

Among the MHSSs that answered the question on the criteria allegedly sufficient to determine whether a bed bug infestation exists, 66% (42/64) cited direct observation of bed bugs, 56% (36/64) mentioned observation of bed bug faeces and blood stains on the sheets, 41% (26/64) thought that an interview with the occupants alone was sufficient and 39% (25/64) cited observation of bite marks on the skin (total >100% due to the possibility of selecting multiple answers).

Among the PMCs, 71% (36/51) cited direct observation of bed bugs, 73% (37/51) mentioned observation of bed bug faeces and blood stains on the sheets, 47% (24/51) thought that an interview with the occupants alone was sufficient and 27% (14/51) cited observation of bite marks on the skin (total >100% due to the possibility of selecting multiple answers).

**Figure 2.**
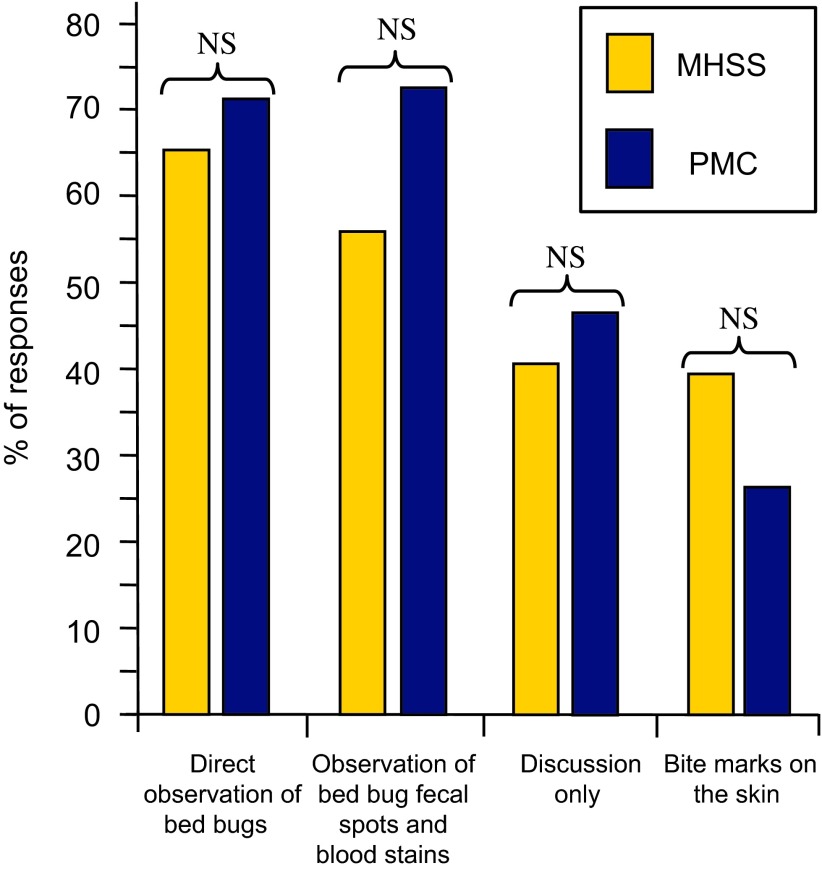
Percentage of responses from Municipal Health and Safety Services (MHSSs) and Pest Management Companies (PMCs) regarding their perception of the criteria thought sufficient for considering a bed bug infestation. Fisher’s exact probability (P) is indicated when significant; NS: Not significant.

A comparison of the answers given by MHSSs and PMCs shows no significant difference between the two (Fig. 2).

### Methods used in practice to determine an infestation

For MHSSs: 66% (42/64) mentioned detailed visual inspection, 42% (27/64) testimony from the occupant, 33% (21/64) observation of bite marks on the occupant’s skin, 17% (11/64) the use of traps (almost always – in 91% of cases (10/11) – in addition to a visual inspection) and 0% the use of a dog (total >100% due to the possibility of selecting multiple answers).

Among PMCs, 96% (49/51) mentioned detailed visual inspection, 53% (27/51) testimony from the occupant, 39% (20/51) observation of bite marks on the occupant’s skin, 27% (14/51) the use of traps (almost always (13/14 cases) in addition to a visual inspection) and 8% the use of a dog (total >100% due to the possibility of selecting multiple answers).

**Figure 3.**
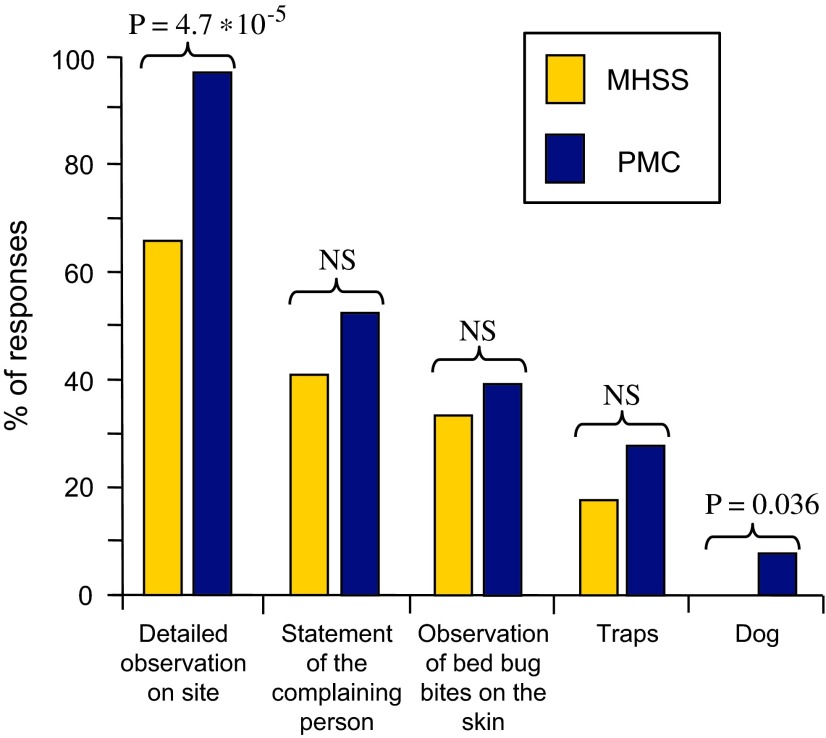
Percentage of responses from Municipal Health and Safety Services (MHSSs) and Pest Management Companies (PMCs) regarding the methods they use in practice to confirm a bed bug infestation. Fisher’s exact probability (P) is indicated when significant; NS: Not significant.

A comparison of the responses given by MHSSs and PMCs indicates significant differences in the responses on detailed visual inspection and dog use (Fig. 3).

### Time spent diagnosing an infestation

**Figure 4.**
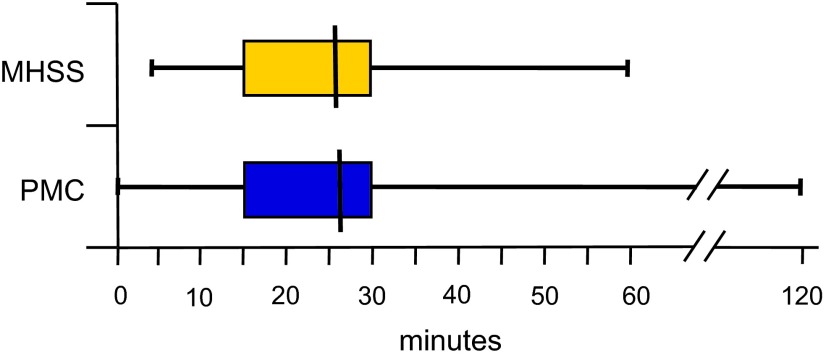
Time taken to confirm the presence of bed bug infestation for Municipal Health and Safety Services (MHSSs) and Pest Management Companies (PMCs). Per category, the 25th–75th percentile is shown by the box plot, the mean value is shown as a line inside the box and the range of values is shown by the lines outside the box.

For MHSSs, the average time necessary to establish a diagnosis is 26.0 minutes (min); the median, 20 min (*n* = 38). For PMCs, the average time necessary to establish a diagnosis is 26.8 min; the median, 25 min (*n* = 50). There is no significant difference between the two averages (Student’s *t* test). For both MHSSs and PMCs, the 25th and 75th percentiles are 15 and 30 min (Fig. 4).

### Attitudes and operational approaches of PMPs to practical control measures

#### Non-chemical control/chemical control

Sixty-seven per cent of PMCs use or recommend a combination of non-chemical (including heat, either dry heat or steam, freezing, vacuum, brushing, etc.) and chemical control (34/51); 31%, chemical means alone (16/51); and 2%, non-chemical means only (1/51).

In the event of control measures being implemented, the number of treatments varies from 1 to 4: 10% of respondents (5/51) say they performed a single treatment, 61% (31/51) two treatments, 4% (2/51) two to three treatments, 24% (12/51) three treatments and 2% (1/51) three to four treatments.

#### Time between treatments

Few responses were received (8). The mean time was 10.1 days; the median, 11; and the range, 2–15.

#### Attitude if no bed bugs observed

Forty-three per cent (22/51) take action to eradicate bed bugs. Fifty-seven per cent (29/51) do not. Those who do take action either carry out a minimal intervention on high-risk areas including bedding (15/51 = 29%) or a full intervention (7/51 = 14%). Those who do not take action await further information from the customer (19/51 = 37%), continue to monitor the property for potential bed bug infestations (7/51 = 14%), set traps (2/51 = 4%) or insist that the customer informs/communicates with his/her neighbours (1/51 = 2%).

#### Post-intervention assessment

Eight per cent (4/51) do not perform such an assessment, 37% (19/51) carry out a post-treatment visit and 63% (32/51) wait to be contacted again by the customer. A minority set traps, be they active (9/51 = 18%) or passive (5/51 = 10%); none uses sniffer dogs (0/51). In the event of a post-treatment visit, the mean number of days between the treatment and said visit is 12.9, the median 15 and the range, 3–21 (*n* = 19). The mean number of visits necessary to resolve a situation involving bed bugs is 2.6, the median is 3 and the range, 1–4 (*n* = 48).

#### Training and information

For MHSSs: 44% (24/55) consider that they are insufficiently informed about the problem of bed bugs, good detection practices and treatments; 62% (34/55) would like specific training; and 65% (36/55) think the general public are not any better informed about or aware of bed bugs than they were 5 years ago.

For PMCs: 19% (9/48) consider that they are insufficiently informed about the problem of bed bugs, good detection practices and treatments; 56% (27/48) would like specific training; and 54% (26/48) think the general public are not any better informed about or aware of bed bugs than they were 5 years ago.

### Salient points from the open comments section

Free-text comments identified some challenges in improving the management of bed bug infestations. We consider useful to report here a selection of opinions that identify weaknesses and opportunities for changes to the current situation.

- The large number of involved stakeholders (general public, travel agencies, international vaccination centres, hospitals, landlords, social housing offices, etc.) is perceived as a difficulty, especially in terms of awareness and mobilisation. Any information, education and communication strategy should take into account this diversity of actors.
- The current training of professionals is regarded as inadequate. Specific training should systematically cover communication to the general public. In this respect, the importance of the human factor is raised with, as an example, the need for considering the motivation of tenants and the well-being of the inhabitants who may be patients, elderly or disabled. This is likely to facilitate or hinder the interventions.
- Besides the existence of poor practices, some respondents underline the marketing of ineffective products and the misinformation of the public by some service providers, especially over the Internet.
- Interventions in collective dwellings constitute an operational and financial challenge exacerbated by a lack of information at social housing offices. In addition, the regulatory framework is inappropriate for intervention in collective dwellings and a desire to see changes and adaptation is reported.

## Discussion

This survey is original in that it actively sought out participants by telephone and/or email. It yielded responses from 119 private and public organisations involved in bed bug control. An important result is that at least one infestation was reported in every single *département* in metropolitan France (96/96) in 2014. To our knowledge, this is the first time such an indication has been available. In addition, there is a perception among respondents that the bed bug problem has grown over the past five years.

This survey provides a variety of responses that permit interesting comparisons between MHSS and PMPs. PMPs, significantly more than MHSS, considered that the bed bug problem is increasing. This point may be related to higher detailed observation on site for diagnostic methods performed and a greater involvement in this issue by PMCs, with regard to MHSS. On the other hand, MHSS and PMPs do not differ in their responses concerning their perception of the diagnostic methods for establishing a bed bug infestation and the time necessary to establish this diagnosis.

Whatever its strengths, our survey also has several limitations. (1) The relatively small number of responses per location precludes a detailed geographical analysis by region, *département* or town, or a quantitative approach. (2) There is no weighting of responses based on the size of the MHSS and PMC concerned. Consequently, a very large company with several hundred employees and a small company with just one employee had the same weight in the survey (one response each). (3) The opinion of the PMP respondent does not necessarily reflect the knowledge or practices of his/her whole PMC. (4) Bias in the PMP sample due to the use of the membership list of a PMC association, whose members tend to be large companies. (5) Low percentage of responding PMCs. (6) Bias in the MHSS sample, possibly leading to under-declaration in the more rural *départements* due to the fact that MHSSs only exist in large urban areas. (7) The results are based on personal statements and are thus reliant on the good faith of the respondent, with no subsequent verification. (8) The potential gap between knowledge of good practices and their actual implementation in the field was not assessed.

The answers are mainly satisfactory, but some are rather disappointing. Especially the notions that an interview with the occupant or bite marks were enough to confirm a bed bug infestation, which are clearly inappropriate methodologies [9]. There are significant percentages of chemical control performed although the presence of bed buds has not formerly been established. In addition, follow-up is not always undertaken after treatment. We should remember that, based on the conclusions of the expert group [5] and the simplified booklet [7], a well-managed approach to bed bug control should comprise six stages: (1) confirmation of bed bug infestation; (2) assessment of the level of said infestation; (3) non-chemical control using various tools and methods such as dry heat, steam, freezing, washing, vacuum, brushing, diatomaceous earth, discarding highly infested items, etc.; (4) if necessary, judicious use of insecticides in two stages 10–15 days apart; (5) assessment of control actions; (6) advice and prevention. The small number of responses to this questionnaire, particularly from PMPs, means that it offers only a partial view of knowledge and practices in this sector. Nevertheless, the approach outlined was followed by only a minority of PMPs and an even smaller minority of MHSSs. An improvement of practices and the introduction of specific training are vital. Yet the training courses for PMPs, which culminate in the award of a biocides certificate, commonly known as “Certibiocide”, last just 3 days and include almost no content on entomology and how to tailor control measures to the specific insects targeted. Professionals and future professionals have few options to be trained on the job, by private companies (e.g. biocide distributors), by colleagues and by paying for specific training on bed bugs. The low response rate for certain questions, such as the time between two treatments, raises questions about knowledge of good practices. Other responses, such as those citing practices for identifying infestations based solely on information provided by customers, are, though rare, clearly contrary to good general insect control practices. It is time to reform and revise the qualifications required for PMPs, perhaps drawing inspiration from practices in countries such as Australia, the USA and Canada, where Best Practice Guides for bed bug control are updated every 2–3 years. More needs to be done to raise awareness among the general public and adapt the regulatory environment if national monitoring of such infestations is to be improved. Integrated control (using both non-chemical and chemical methods) and collaboration between the pest controller and the customer are the keys to success. This collaboration often has little formal structure. In the midst of all this, we should never forget that the occupant whose home is infested is tired and stressed and is incurring unplanned expenses. All these factors make control more difficult.

The present survey confirms the perception that bed bugs are a nuisance that is growing fast. This increase has already been confirmed in North America, Europe and Australia [8, 15, 17]. In order to document it in metropolitan France, we could take inspiration from the model adopted in Montreal (*Règlement 03-096 sur la salubrité*, *l’entretien et la sécurité des logements*, Ville de Montréal, 2012), for example, with an obligation for insect control companies to declare their interventions to the municipality concerned, providing simple information such as the date, the town, the neighbourhood and the semi-quantitative infestation level. In the medium term, this would make it possible to monitor increases or decreases in infestations at the national level, and thus to determine whether improvements are being made through good management of bed bug control, or, conversely, whether the situation is worsening in an alarming manner.

Our knowledge of the distribution of bed bugs in metropolitan France remains patchy, and even more so for France’s overseas territories. Collection of entomological data is clearly insufficient in both qualitative (presence/absence) and quantitative terms. The establishment of a national database would make it possible to continue the approach initiated in this study. The goal would be to formalise feedback on the presence of bed bugs, particularly from PMPs. Basic information about this observatory could be provided during training under the Certibiocide scheme.

Furthermore, it would appear to be both difficult and desirable to assess the impact of bed bug control activities on the level of insecticide resistance in bed bug populations.

In conclusion, this survey found that bed bug control is present in all *départements* of metropolitan France. Bed bug control is primarily managed by private Pest Management Professionals (PMPs). The local public bodies (MHSSs) are often called upon, but are rarely responsible for control operations. Although some firms specialise in bed bug control and do so very efficiently, there appears to be a significant lack of information and training about bed bugs and the methods for controlling them, for MHSSs, PMCs and the general public. This lack of information makes effective management of bed bug control difficult.

## Supplementary Material

Additional file-Questionnaire Punaises de lit
