# Supplementary material for: The Common bed bug (Cimex lectularius) in metropolitan France. Survey on the attitudes and practices of private- and public-sector professionals
Source: Parasite. 2016 Sep 8;23:38. doi: 10.1051/parasite/2016038 (PMC5018931; doi:10.1051/parasite/2016038)
Supplement: Additional file-Questionnaire Punaises de lit [file parasite-23-38-s1.pdf]

La Punaise de lit
Questionnaire national à l'attention des SCHS et des Opérateurs 3D

Les punaises de lit (*Cimex lectularius* et *Cimex hemipterus*) avaient quasiment disparu du territoire français métropolitain depuis les années 1950, en lien avec l'amélioration de l'hygiène de l'habitat et peut-être aussi en lien avec l'utilisation domestique du DDT.
Une recrudescence de ces punaises semble être observée à partir des années 1990 dans la plupart des pays développés, sans qu'aucun élément tangible ne vienne appuyer cette hypothèse. Quoiqu'aucune transmission de pathogènes à l'homme par *Cimex* n'a jusqu'à présent été démontrée, cette espèce pose un problème de nuisance, qu'il convient dans un premier temps d'évaluer, afin de mettre en place les mesures de gestion les mieux adaptées.

Dans cette perspective, un groupe de travail du CNEV (Centre National d'Expertise sur les Vecteurs) vous soumet ce questionnaire.

Ce questionnaire s'adresse aux SCHS et aux professionnels de la désinsectisation, qui, en contact direct avec le terrain, sont les mieux placés pour fournir des éléments permettant de caractériser la situation et la pratique de la lutte. Ce questionnaire est conçu pour être rempli en 10 minutes.

VOUS

1. Personne répondant au questionnaire

Nom/Prénom

Organisme/Société/Institution

Adresse

Mail

Téléphone

2. Si vous êtes un opérateur 3D, votre entreprise

- Année de création :
- Nombre de personnes employées :
- Nombre d'interventions annuelles (tous insectes confondus) :
  - ☐ <10
  - ☐ 10-50
  - ☐ 50-100
  - ☐ 100-200
  - ☐ >200

- Zone d'intervention : Mentionner les départements

3. VOS ACTIVITES « PUNAISES DE LIT »

3.1. Avez-vous été sollicités pour des interventions avec un plaignant mentionnant les punaises de lit ?

- ☐ Oui
- ☐ Non

3.2. Avez-vous réalisé des interventions contre punaises ?

- ☐ Oui
- ☐ Non

3.3. Si oui, depuis combien d'années environ ? ☐

3.4. Quelle proportion d'interventions cela représente-t-il environ au sein de votre activité ?

- ☐ Moins de 20%
- ☐ de 20 à 40%
- ☐ de 40 à 60%
- ☐ de 60 à 80%
- ☐ plus de 80%

3.5. Combien d'interventions contre les punaises de lit réalisez-vous chaque année (une intervention est ici considérée comme une ou plusieurs interventions au sein d'un même immeuble ou hôtel) ?

- ☐ Aucune
- ☐ 1 à 10
- ☐ 11 à 50
- ☐ 50 à 100
- ☐ 100 à 200
- ☐ plus de 200

3.6. Si vous disposez d'une base de données relative à vos interventions punaises, accepteriez-vous de nous communiquer le nombre d'interventions par commune (afin de mieux apprécier l'étendue de ce problème au niveau du territoire) ?

- ☐ Nous n'avons pas de base de données
- ☐ Nous acceptons de communiquer ces informations
- ☐ Nous ne souhaitons pas communiquer ces informations

3.7. Au cours des 5 dernières années, le nombre de vos interventions contre les punaises de lit :

- ☐ A augmenté
- ☐ A diminué
- ☐ Est resté stable

VOS PRATIQUES

4. Diagnostic

4.1 Parmi les critères suivants, lesquels vous semblent suffisants pour statuer sur l'infestation par des punaises de lit?

- ☐ Demande du client
- ☐ Entretien détaillé avec le client : o/n
- ☐ Observation directe de punaises : o/n
- ☐ Observation de traces (déjections de punaises, tâches de sang…) : o/n
- ☐ Observation de traces de piqûres caractéristiques sur la peau : o/n
- ☐ Autre (précisez)

4.2 Quelle méthode utilisez-vous pour diagnostiquer la présence de punaises de lit dans un logement (plusieurs choix possibles) ?

- ☐ Aucune, j'interviens suite à une demande client
- ☐ Inspection visuelle
- ☐ Chien détecteur
- ☐ Pièges (passifs ou actifs)
- ☐ Piqures du client
- ☐ Autres (préciser)

4.3 Quelle est la durée moyenne de votre visite pour établir un diagnostic pour un logement ?

4.4 En cas de présomption non confirmée de présence de punaises de lit, quelle(s) suite(s) donnez-vous ?

- ☐ Attente de nouvelles informations par le client
- ☐ Intervention minimale aux points à risque (literie)
- ☐ Intervention complète
- ☐ Autre (précisez)

5. Contrôle

5.1 Quelle(s) méthode(s) de lutte utilisez-vous ou recommandez-vous contre les punaises de lit ?

- ☐ Lutte chimique
- ☐ Lutte mécanique
- ☐ Les deux

5.2 En cas de traitement insecticide, vous effectuez généralement :

- ☐ Un traitement unique: o/n
- ☐ Deux traitements (si oui à combien de jours d'intervalle) : o/n
- ☐ Autres : précisez

5.3 En cas de traitement insecticide, quel(s) produit(s) biocide(s) (appellation commerciale) utilisez-vous régulièrement : Précisez

5.4 Comment évaluez-vous a posteriori l'efficacité des opérations de lutte ?

- ☐ Nouvelle visite post-traitement (si oui, combien de temps après) : o/n
- ☐ Pose de pièges attractifs (moniteurs) (si oui, combien) : o/n
- ☐ Pièges passifs: o/n
- ☐ Attente d'informations par le client: o/n
- ☐ Chien détecteur: o/n
- ☐ Autre

5.5 En moyenne, combien de visites sont nécessaires, depuis le diagnostic jusqu'à la résolution du problème ?

5.6 Champ libre pour des commentaires relatifs au contrôle :

6. VOTRE AVIS SUR LA FORMATION / INFORMATION

6.1 Estimez-vous être assez informé sur la problématique des punaises de lits, les bonnes pratiques de détection, et les traitements ?

- ☐ Oui
- ☐ Non
- ☐ Ne sais pas

6.2 Seriez-vous intéressés par une formation spécifique sur ces sujets ?

- ☐ Oui
- ☐ Non
- ☐ Ne sais pas

6.3 Estimez-vous que le grand public est mieux informé et sensibilisé aux punaises de lits qu'il y a 5 ans ?

- ☐ Oui
- ☐ Non
- ☐ Ne sais pas

7. CHAMP POUR DES COMMENTAIRES LIBRES
